# The clustering of physical activity and screen time behaviours in early childhood and impact on future health-related behaviours: a longitudinal analysis of children aged 3 to 8 years

**DOI:** 10.1186/s12889-022-12944-0

**Published:** 2022-03-21

**Authors:** Rosemarie Martin, Joey Murphy, Daniel Molina-Soberanes, Elaine M Murtagh

**Affiliations:** 1 Department of Reflective Pedagogy and Early Childhood Studies, Mary Immaculate College, University of Limerick, Limerick, Ireland; 2 Centre for Exercise, Nutrition and Health Sciences, School for Policy Studies, University of Bristol, BS8 1TH Bristol, UK; 3 Department of Preventive Medicine and Public Health, University of Granada, 18016 Granada, Spain; 4 Department of Physical Education and Sport Sciences, University of Limerick, Limerick, Ireland

**Keywords:** Physical activity, Screen time, Children, Cluster analysis, Longitudinal

## Abstract

**Background:**

Meeting physical activity and screen time guidelines has been associated with improved health in children. Research has shown that lifestyle behaviours happen in combination and can be tracked into later life. Thus, a complex approach is needed to identify the effects of physical activity and screen time altogether. This study aims to identify clusters of both behaviours in a cohort of Irish 3-year-old children (*n* = 8833) and determine the association with sociodemographic characteristics and behaviours at age 5 and 7-8.

**Methods:**

Data from the “Growing Up in Ireland” study collected between 2010 and 2016 was used in this study. Two-step cluster analysis was used to understand how physical activity and recreational screen time behaviours group together among 3-year-old children. Binary logistic regressions were conducted to examine if cluster placement at age 3 determined physical activity and recreational screen time behaviours at age 5 and 7-8 years, while controlling for gender of child, gender, age and employment status of the primary caregiver.

**Results:**

Six clusters were identified in 9771 (49.3% female) 3-year-old children with the majority falling into a “High Active & Mixed Screen Time” (23.2%). Those in the “High Active & Mixed Screen Time” cluster at age 3 were more likely to engage in all physical activities reported at age 5 (*p* < 0.01) and age 7-8 (*p* < 0.01) when compared to a “Low Active & Screen Time Exceed” cluster. Children categorised in a “Moderate Active & Screen Time Below” and “Moderate Active & Screen Time Exceed” were more likely to engage in the same physical activities at age 5 and 7-8 (*p* < 0.05 - *p* < 0.01). However, children in the latter cluster were also more likely (*p* < 0.05) to play on a computer or tablet device.

**Conclusions:**

This paper highlights the importance of establishing positive health-related behaviours during early childhood, as this predicts future engagement in health-promoting activities. Regardless of screen time level, being part of a cluster with moderate or high levels of physical activity positively influences a child’s future physical activity at age 5 and again at age 7 -8 years. The multiple layers of influence on a child’s development should be leveraged to support the adoption of health-enhancing behaviours.

**Supplementary Information:**

The online version contains supplementary material available at 10.1186/s12889-022-12944-0.

## Background

The benefits of physical activity (PA) on children´s health are strongly evidenced, and comprise positive impacts for cardiorespiratory and muscular fitness, bone health, mental health, and weight status [1]. To achieve these benefits, the World Health Organization (WHO) recommends 180 min of daily PA of which at least 60 min are of moderate-to-vigorous intensity (MVPA) for pre-schoolers [2], and it suggests at least 60 min of MVPA daily for children aged 5-years or older, regardless of their total amount of PA [3]. Unfortunately, only 20% of youth are meeting the WHO recommendations worldwide [3]. There is a universal concern about low rates of PA in children of all ages, despite the fact that PA levels can vary greatly among different countries [4]. In Ireland, studies addressing PA levels of children have usually focused on those aged 9-years or older [5, 6].  Nevertheless, two different databases which include children under 9-years have shown that most of them do not meet the WHO recommendations, with 59% of children aged 5-years or older, and 78% of 8- to 11-year-olds reported as insufficiently active [6].

There are several domains of PA during childhood, such as active commuting to school – either walking or cycling, Physical Education classes, organised sports participation and unstructured active play during free time, especially in pre-schoolers [7]. In fact, active play has been associated with both higher levels of total PA and higher intensity levels among school-aged children [8]. Moreover, active play during free time can offer more than 50% of total PA in children aged 5-7 years and is associated with achieving a greater number of days with at least 60 min of MVPA [7]. Parental support for this healthy behaviour is important during childhood [9]. This can be provided through direct involvement in children´s active play comprising a wide range of activities and intensities, or as logistic support for participation in organised sports [10]. In Ireland, only 22% of parents reported direct co-participation in their children´s daily PA through sports or active play [6]. Parents´ PA has even been found to influence their children´s PA into middle age as they become role models for their grown up children [11].

The National Physical Activity Plan for Ireland suggests a coordinated approach to increase PA across the whole population based on school and community settings [12]. It is essential to initiate these strategies at an early age because PA can be tracked throughout the lifespan, from pre-schoolers to grade-schoolers, to adolescents, and to young adults, as shown by a 27 year-follow-up study [13]. Despite that, previous meta-analysis has pointed out a decrease of 13% of MVPA in the transition from adolescence into early adulthood [14]. Recent studies have suggested that this declination in PA can be identified earlier [15], as soon as in 7-year-old children [16], with a decrease of 6 min per day of MVPA each year during childhood [17]. Furthermore, this reduction in PA can be accompanied by an increase in sedentary behaviours [18].

Among the domains of sedentarism, passive recreational screen time (ST) has shown the strongest association with risk factors for children´s health [19]. As with physical activity, ST can be tracked from early childhood, to grade-schoolers, to youth, and to young adulthood [20], and its rates are alarming. Several Irish studies have highlighted the significant number of children including pre-schoolers who did not meet international recommendations of less than 60 min passive ST daily. The prevalence of non-compliance reported in the studies ranges from 44% to over 90% [21–24]. Unfortunately, even though WHO recommends no more than 1 h of passive ST daily for pre-schoolers [2], there is not a widely accepted threshold for children aged 5 or older, as there is not enough evidence to establish a precise cut-off point on how much passive ST should be tolerated. What is clear is that passive ST should be limited and discouraged, as stated by the WHO guidelines [2] on PA and sedentary behaviour [2]. Literature suggests that increasing PA with limited ST is associated with the best health-related quality of life, and deceasing PA with increased ST seen as the worst [25]. These findings highlight the importance of disentangling how children spend their time in a 24-hour period, with special emphasis on MVPA as even though it probably comprises the least amount of time spent on movement behaviour, it likely contributes with the greatest impact on children´s health. In fact, compositional analyses have been used to address this issue, and their results reinforce the key role of MVPA, especially for overweight and obese children [26]. Still, complex approaches are needed to identify the effects of PA and ST in combination, as the two behaviours are associated with health outcomes [1] and therefore their impact may overlap.

There can be a myriad of factors related to PA or physical inactivity throughout lifespan [27]. Identifying groups of people who share similar lifestyle behaviours is an opportunity to optimize resources targeting public health concerns [28]. Some recent efforts have been made, in a variety of settings, to identify such groups. In their systematic review, Parker et al. [29] found 36 studies addressing the sociodemographic characteristics and other correlates of activity-related behavioural typologies among adolescents. They observed consistent results grouping adolescents with high levels of PA and low levels of sedentary behaviours, and also with low levels of PA and high levels of sedentary behaviours. The authors report that the latter group was often associated with other risk-related behaviours. However, little is known about the clustering of activity-related behaviours in younger children, as although they are not absent, these approaches are scarce in this population. A recent longitudinal study followed children aged 6- to 11-years, over a three-year period [30]. The researchers conducted latent class analysis based on self-reported data to elucidate different activity profiles at baseline, and linear mixed growth models were then used to assess longitudinal changes in the children’s behaviours. The authors identified three groups at baseline, “social screenies”, “excercisers”, and “non-sporty active commuters” and they reported that trends in MVPA and ST behaviours remained similar at follow up [30]. However, it must be noted that participants under 8-years-old were excluded from the study due to their limitations in providing valid and reliable information [30], thus highlighting the challenges in correctly identifying behavioural groupings in younger children. This combined with the evidence that movement behaviours track across life stages [17, 30] emphasize the need to identify and evaluate such clusters in this age group.

Cluster analysis is another statistical approach that can classify participants into homogenous groups among different categories of lifestyle behaviours either healthy or unhealthy [31]. Assessing pre-schoolers and grade-schoolers’ behaviours, such as PA and ST, in a cluster analysis from a large longitudinal data set could help in understanding the complexity of their relationship and how they relate to lifestyle behaviours. Thus, the aim of this paper, using data from the *Growing Up in Ireland* study, was to identify the clustering of PA and ST in a cohort of Irish children aged 3 years, and to determine any association with sociodemographic and lifestyle characteristics related to their PA and ST at age 5 and 7- 8 years.

## Methods

The current study analyses waves two, three and four of the Infant Cohort data collected as part of *Growing Up in Ireland*, *The National Longitudinal Study of Children* (GUI). GUI synchronises with other contemporary longitudinal child cohort studies such as the Millennium Cohort study (UK), Growing up in Australia, the National Longitudinal survey of children and youth (Canada) and the Early Childhood Longitudinal Study (US) [32]. The large, nationally representative data collected for GUI has enabled robust analysis of the many factors that may influence the development of children. Details outlining the Infant Cohort study design, instrumentation used, and procedures adopted for each wave of data collection have been published in the GUI Technical Reports [33–35]. The Infant Cohort is a nationally representative sample of 11,134 infants. The sampling frame consisted of all children born between 1st December 2007 and the 30th of June 2008, who were on the Child Benefit register in the Republic of Ireland (*n* = 41,185). The sample was randomly selected on a systematic basis, pre-grouped by parents’ marital status, nationality, county of residence and number of children in the benefit claim. Data for 11,134 infants aged 9 months, and their primary caregiver (PCG) were available for analysis in Wave 1. Being a longitudinal study, this cohort of infants and their PCG were the target sample for subsequent waves when the study children were 3 years old, 5 years old, and 7- 8 years old (children were aged 7 or 8 during this wave due to the timing of data collection). The second wave of data was collected between December 2010 and July 2011 (*n* = 9,793). The third wave of data collection occurred between March and September 2013 (n= 9,001) and Wave four data were collected between March and April 2016 (*n* = 5,344). A dedicated Research Ethics Committee established by the Department of Health provided ethical approval for the GUI study [33]. Primary caregivers provided written informed consent to participate in the study.

The conceptual framework underlying GUI draws heavily from Bronfenbrenner’s bio-ecological model (1993) and is outlined in previous literature [36]. In summary, the child is placed at the core of the framework and their world is a multi-layered set of concentric and interconnected environmental systems which influence their development. The GUI study focuses on a broad range of internationally recognised child outcomes to establish how well children in Ireland are developing across three main domains: (1) Socio-emotional, behavioural and family (2) Educational/ cognitive and (3) Health [35]. In the first three waves, these outcomes were assessed using data collected in a face-to-face interview with the study child’s PCG and his/her partner/spouse (where applicable), in the family home. Wave four data were collected via a short postal questionnaire self-completed by the study child’s PCG only [34, 35].

A response rate of 64% of all PCGs approached to participate was achieved for Wave 1 (*n* = 11,134) [37]. Questionnaires were completed by *n* = 9,793 PCGs at Wave 2 representing a response rate of 88%. The current study includes data from Waves 2, 3, and 4 which correspond to timepoints 1, 2, and 3 respectively. Time 1, Time 2 and Time 3 will be used to refer to the corresponding waves for the remainder of this paper. This study includes data from respondents that participated at Time 1 and at least one later time point. 90% (*n* = 8,833) of Time 1 respondents also participated in at least one later timepoint so were included in the current study. 52% of Time 1 respondents participated at all 3 timepoints (*n* = 5,086). Of the Time 3 respondents (*n* = 5,344) 95% had participated in all previous timepoints. Respondents that participated at Time 2 and/or Time 3 but not at Time 1 were excluded from analysis in this study (*n* = 329). Reweighting of the GUI data was carried out by adjusting the distribution of the sample using the number and characteristics of infants and their families in the 2006 Census of Population and the 2008 Child Benefit Register [38].

### Measures

Different self-report measures were used at each of the 3 time-points. Certain responses were recategorised for ease of understanding and use with later statistical tests.

- Time 1 (3 years of age).

Demographic information including the gender of the child, and the gender, age and employment status of the PCG were collected using multiple choice questions at this stage. Primary caregiver self-reported measures assessing PA co-participation, free time, use of a bicycle or scooter and ST were used. Co-participation in PA was measured by asking “On how many days in an average week does anyone at home play active games with this child (e.g. football)”, with responses categorised as “0-2 days”, “3-5 days”, “6-7 days”. Free time play was measured by asking “What does this child prefer to do when he/she has a choice about how to spend free time”, with responses categorised as “Usually chooses active pastimes”, “Usually chooses inactive pastimes”, “Both active or inactive pastimes”. Use of a bicycle or scooter was measured by asking “Can your child ride a tricycle or other similar toy vehicle with pedals”, with responses categorised as “Yes (e.g. uses pedals or pushes along without using pedals)” or “No (e.g. does not use or does not have a tricycle)”. ST was measured by asking “Typically, how many hours a day does this child sit and watch television or videos/DVDs?” with responses collected in hours and minutes. Responses were dichotomised into meeting or exceeding the ST recommendations set out by the Health Service Executive (e.g. no more than 60 min a day) [39].

- Time 2 (5 years of age).

Demographic information including the gender, age and employment status of the PCG were collected using multiple choice questions at this stage. Self-report measures assessing sport club or group participation, climbing (e.g. trees, frame, wall bars), playing with a ball, playing chase, riding a bicycle, tricycle or scooter, skating and ST were used. Sports club/group participation was measured by asking “Does this child attend a sports club or sports group”, with responses categorised as high (2 or more hours per week), moderate (1 h per week), and low (less than once a week). Other behaviours were measured by asking “Looking at card E6, can you tell me how often this child <insert activity>”, with responses categorised as high (more than 2 times a week), moderate (1-2 times a week), and low (less than once a week). Finally, ST was assessed by asking “Can you tell me how often the study child takes part in the following activities outside school: playing on a device like a computer or iPad by themselves” with responses categorised as high (more than 2 times a week), moderate (1-2 times a week), low (less than once a week).

- Time 3 (7- 8 years of age).

Demographic information including the gender and employment status of the PCG were collected using multiple choice questions at this stage. Self-report measures assessing participation in activities outside of school and ST were used. Activities outside of school were measured by asking “Can you tell me how often the study child takes part in the following activities outside school”, with responses categorised as high (more than 2 times a week), moderate (1-2 times a week), low (less than once a week). The activities included playing games that involve a lot of running around, playing games that involve some activity, riding a bicycle, tricycle or scooter, playing on a device like a computer or iPad by themselves, and enjoying dance, music and movement.

### Statistical analysis

SPSS for Statistics (version 25; SPSS Inc, Chicago, IL) was used for all analyses. Those who did not complete all the items needed for the cluster analysis were removed from the study. Descriptive statistics were calculated for demographic data and each of the behaviours included in the cluster analysis. As stated in the introduction, several behaviours influence future engagement in PA behaviours. For this reason, a two-step cluster analysis was used as an explanatory tool to identify specific behavioural clusters. This method is designed to handle large data sets and enables the inclusion of categorical data [40]. The number of clusters was based on the log-likelihood distance and Schwarz Bayesian criterion (BIC) [40]. Where the BIC is found to reduce continuously as the number of clusters increase, the ratio of BIC Changes and ratio of Distance Measures were assessed to find an acceptable cluster model [41]. This is done since the most acceptable model, becomes difficult to interpret due to the increased complexity of additional clusters [42]. To assess the reliability of the clusters, the analysis was repeated in an internal sample of 50% of the total study sample and a kappa statistic was used [43]. This approach has been used in the past to understand health-related behaviours of Irish university students [31].

Once a valid and reliable cluster grouping had been identified, a series of binary logistic regression tests were performed to explore the influence of cluster placement at 3 years of age on the future behaviours of children at age 5 years and age 7- 8 years. Logistic regression analysis allows for categorical and continuous scaled variables to predict any categorical scaled criterion [44]. A binary logistic regression was performed for each of the PA behaviours at age 5 and age 7- 8 years exploring the likelihood of children to be categorised as “moderate” or “high” as opposed to “low”. Gender of the child, and gender, age (except for age 7 or 8 as data was unavailable), and employment of the primary care giver was controlled for in the analysis. The “Low” category for each behaviour and the “Low Active & High ST” cluster were used as the reference category for all analysis. The reference category for the dependent variable (i.e. behaviours) was always set as “Low” as we wanted to assess the increased likelihood of participating in future behaviours. The reference category for the independent variable (i.e. clusters) was set as “Low Active & High ST” as it was seen as the least preferred combination. This allowed us to assess if being a member of a “healthier” cluster (e.g. more PA or less ST) meant being more active at follow-up. The numbers used in each regression analysis are lower in some instances due to less participants completing that measure at future time-points. Results are presented as odds ratios (OR) and 95% confidence intervals, with percentages used in-text.

## Results

After data cleaning, the analytical sample at time 1 comprised 9,771 parents (98.4% female) who responded regarding their child (49.3% female; age = 3 years). The number of responses reduced at time 2 (*N* = 8695) and time 3 (*N* = 5075) with the majority of PCGs identifying as female at all timepoints (>97%). As expected, the majority of PCGs reported being older at time 2 compared to time 1. Data regarding age at time 3 was unavailable. Most PCGs reported being in work or training across all timepoints (55.8 – 64.7%) and taking care of home duties (25.8 - 34.8%). A small proportion of PCGs (1.7% and 2.7%) noted that they were not the same respondent as the previous timepoint at time 1 and time 2, respectively. Table 1 provides detailed demographic information regarding the PCGs included at each timepoint. More background information regarding the children included in the study and their PCGs has been published previously and can be found in the Infant Cohort Reports [34, 45, 46].

**Table 1:** Demographic characteristics of primary caregivers included at each timepoint

	Time 1 (N; %)	Time 2 (N; %)	Time 3 (N; %)
**Total Number**	9771	8695^a^	5075^ab^
Gender			
Male	159 (1.6)	178 (2.0)	113 (2.2)
Female	9612 (98.4)	8517 (98.0)	4962 (97.8)
Age			
18-29 years	1839 (18.8)	935 (10.8)	DNA
30-39 years	6299 (64.5)	5038 (57.9)	
40+ years	1633 (16.7)	2722 (31.3)	
Employment Status			
Unemployed	526 (5.4)	374 (4.3)	DNA
At school or education	180 (1.8)	124 (1.4)	3244 (1.8)
At work or in training	5457 (55.8)	5057 (58.4)	3244 (64.7)
Home Duties	3397 (34.8)	2846 (32.9)	1295 (25.8)
Other	211 (2.2)	257 (3.0)	382 (7.6)
Different respondent from previous timepoint		145 (1.7)	141 (2.7)

*DNA* Data Not Available; ^a ^less responses for employment status; ^b ^less responses for different person from previous timepoint

Table 2 displays the clusters identified from the behaviours at time 1. Six clusters were identified from the analysis based on observation of the models auto-generated through SPSS. A model of six clusters was selected as the BIC was lower than models with less clusters, had a reasonably high ratio of BIC change (0.34) and ratio of Distance Measures (1.072), and deemed to be interpretable. Furthermore, the selected model was found to have excellent reliability (kappa = 1.00) with the internal sample of 50% signifying the consistency of the clusters in this population. Within each cluster, the reported engagement of the child in (1) PA co-participation, (2) free time activities, (3) use of a bicycle, and (4) ST behaviours (based on the HSE guidelines [39]) is presented. Each cluster was given a descriptive name based on the behaviours identified within them. A breakdown of the child’s gender, PCG gender, age, and employment status for each cluster identified at time 1 is available in Supplementary File 2.

**Table 2:** Cluster characteristics based on various behaviours

Cluster No.	1	2	3	4	5	6	Total
**No. Participants (%)**	1951 (20)	2263 (23.2)	892 (9.1)	1558 (15.9)	1139 (11.7)	1968 (20.1)	9771
PA co-participation							
0-2 days	1041	0	187	0	0	0	1228
3-5 days	541	0	373	1558	586	992	4050
6-7days	369	2263	332	0	553	976	4493
**Free Time**							
Usually Inactive	1178	0	203	0	0	0	1381
Usually Active	351	2263	308	1558	0	0	4480
Both	422	0	381	0	1139	1968	3910
**Use a Bicycle**							
Yes	1951	2263	0	1558	1139	1968	8879
No	0	0	892	0	0	0	892
**ST Guidelines (60 min)**							
Below	685	1010	288	635	1139	0	3757
Exceeding	1266	1253	604	923	0	1968	6014
**Descriptive name for cluster**	Low Active & ST Exceed	High Active & Mixed ST	Mixed activity, No bike & ST Exceed	Mod Active, Active FT & ST Exceed	Mod Active & ST Below	Mod Active & ST Exceed	

*ST* Recreational Screen Time

### Influence of demographic characteristics and cluster placement (at age 3) on PA behaviours at age 5

**Fig. 1:**
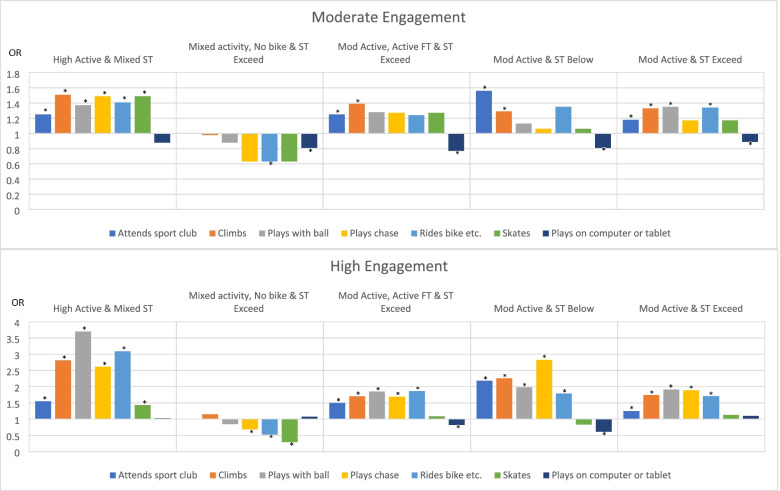
Likelihood of engaging in various activities at age 5 when placed in any cluster compared to the Low Active & ST Exceed cluster at age 3. Legend: * = *p* < 0.05, ST = Recreational Screen Time, Mod = Moderate, FT = Free Time, OR = odds ratio

Figure 1 shows the increased likelihood of engaging in various activities at age 5 when placed in any cluster compared to the “Low Active & ST Exceed” cluster at age 3. Full details of the regression models and results, including odds ratios and 95% confidence intervals can be found in Supplementary File 3. All regression models were significant for differences between low and moderate, and low and high engagement in each activity at 5 years of age. Boys were 11–43% more likely than girls to attend a sports club, 22–65% more likely to engage in climbing activities, 40% to three times more likely to play with a ball, 19–45% more likely to play on a computer or tablet, but 58% less likely to play chase or ride a bike, 32–23% less likely to ride a tricycle or scooter and 58–63% less likely to engage in skating activities. The age category and employment status of the PCG had varying influence on the children’s activities reported. Compared to the reference category (i.e. “Low Active & ST Exceed”), children who were members of the “High Active & Mixed ST” cluster at 3 years of age, were 25–51% more likely to engage in all activities moderately and 43% to 3.7 times more likely to engage highly in all activities at age 5. Children categorised in the “Mixed activity, No bike & ST Exceed” cluster at 3 years of age were 31% less likely to play chase, 37–57% less likely to ride a bicycle, tricycle or scooter, 71% less likely to skate, and 19% less likely to play on a computer or tablet at age 5. Children categorised in the “Mod Active, Active FT & ST Exceed” cluster at age 3 were 25–50% more likely to attend a sports club, 39–70% more likely to engage in climbing activities, 85% more likely to play with a ball, 69% more likely to play chase, 86% more likely to ride a bike, tricycle or scooter, and 18–23% less likely to play on a computer or tablet at age 5. Children categorised in the “Mod Active & ST Below” at 3 years of age were 56% to 2.2 times more likely to attend a sport club, 29% to 2.3 times more likely to engage in climbing, 98% more likely to play with a ball, 2.8 times more likely to play chase, 79% more likely to ride a bike, tricycle or scooter, and 19–39% less likely to play on a computer or tablet at age 5. Children categorised in the “Mod Active & ST Exceed” cluster at 3 years of age were 18–25% more likely to attend a sports club, 33–74% more likely to engage in climbing activities, 35–91% more likely to play with a ball, and 89% more likely to play chase, 34–71% more likely to ride a bike, tricycle or scooter at age 5.

### Influence of demographic characteristics and cluster placement (at age 3) on PA behaviours at age 7- 8

**Fig. 2:**
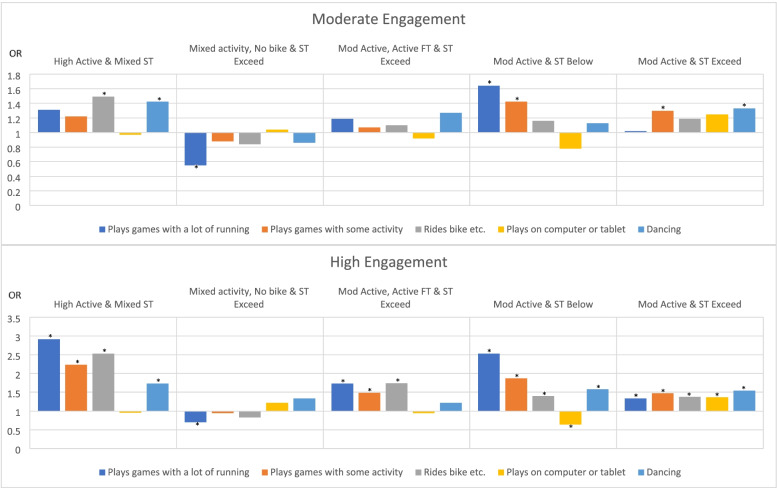
Likelihood of engaging in various activities at age 7-8 when placed in any cluster compared to the Low Active & ST Exceed cluster at age 3. Legend: * =*p* < 0.05, ST = Recreational Screen Time, Mod = Moderate, FT = Free Time, OR = odds ratio

Figure 2 shows the increased likelihood of engaging in various activities at age 7 or 8 when placed in any cluster compared to the “Low Active & ST Exceed” cluster at age 3. Full details of the regression models and results, including odds ratios and 95% confidence intervals can be found in Supplementary File 4. All regression models were significant for differences between low and moderate, and low and high engagement in each activity at age 7 or 8. Boys were 2 times more likely to play games with a lot of running, 85% to 2.5 times more likely to play on a computer or tablet device, but 31–36% less likely to engage in games with some activity, 27–30% less likely to ride a bicycle, tricycle or scooter and 82–93% less likely to enjoy dance, music and movement. Children were 44% less likely to highly engage in games with some activity where the primary care giver was categorised as male. As at age 5 years, the employment status of the primary care giver had varying influence on the children’s activities reported. Compared to the reference category (i.e. “Low Active & ST Exceed), children who were members of the “High Active & Mixed ST” cluster at 3 years of age, were 2.9 times more likely to play games with a lot of running, 2.2 times more likely to play games with some activity, 49% to 2.5 times more likely to ride a bicycle etc., and 42–73% more likely to enjoy dancing, music and movement at age 7 or 8. Those in the “Mixed activity, No bike & ST Exceed” at age 3 were 30–45% less likely to play games with a lot of running at age 7 or 8, while those in the “Mod Active, Active FT & ST Exceed” cluster at age 3 were 73% more likely to play games with a lot of running, 48% more likely to play games with some activity, and 74% more likely to ride a bicycle etc. at age 7 or 8. Compared to the reference category, those in the “Mod Active & ST Below” and the “Mod Active & ST Exceed” clusters at age 3 were 34% to 2.5 times more likely to engage in high levels of all activities reported except for playing on a computer or tablet device at age 7 or 8. For this activity, those categorised in “Mod Active & ST Below” were 36% less likely and those in “Mod Active & ST Exceed” were 37% more likely to play on a computer or tablet device.

## Discussion

This study set out to identify the clustering of PA and ST in a cohort of Irish children aged 3 years, and to determine any association with sociodemographic and lifestyle characteristics related to their PA and ST at age 5 and 7- 8 years. One previous study examined the clustering of PA and sedentary behaviour in children (aged 8-11 years) and adolescents (aged 11-18 years) over a three-year period [30] However, to our knowledge, this is the first longitudinal paper to examine the clustering of PA and ST behaviours in children as young as 3 years old. This is a crucial period in childhood as it encompasses the transition from pre-school to primary school, bringing about significant changes in children’s educational and recreational circumstances which may impact on their PA levels [47].

As illustrated in Table 1 which outlines the behaviours of the children at 3 years old, 46% were usually active, 14% were inactive, while 40% engaged in a mix of both active and inactive activities during their free time. In their review of PA and sedentary time among pre-school children internationally, O’ Brien et al. (2018) report that children aged 2-5 spend a significant amount of time engaged in sedentary behaviours [48]. It is difficult to compare the children’s PA levels to existing research due to variance in results. The challenge of reporting the prevalence of PA among this age group has been previously acknowledged in the literature [48, 49]. The Global Matrix 3.0 [4] reports that of the 20 countries which included data for children aged 5- 17 years engaging in unstructured/unorganised active play, the average prevalence was 34-39%. This illustrates that children in the current study were performing slightly better than children and youth internationally for free-time PA. The majority of children in the current study (87.5%) engaged in moderate to high levels of PA co-participation with their PCG (i.e. 3-7 days/week). This exceeds levels of PA co-participation reported in previous international research. Dunton et al. (2012) and Hnatiuk et al. (2017) report that the majority of PCGs rarely participate in PA type behaviours with their child [50, 51]. These studies found that PCGs and their children engaged in more sedentary activities together than active ones. Over 60% (61.5%) of the children in the current study exceeded the ST recommendations as set out by the HSE [33]. This is synonymous with international research which indicates that the majority of similar aged children exceed 60 min of ST per day [52, 53].

Results of our study reveal that regardless of being below or exceeding the ST limits of 60 min per day [39], being part of a cluster with moderate or high levels of activity influenced the child’s future PA at age 5 and at age 7- 8. Children in moderate and high active clusters at age 3 were likely to attend a sports club/group, climb on trees/frames/wall bars, play with a ball, play chase, ride a bike/tricycle/scooter at age 5 regardless of whether they exceeded 60 min of ST per day or not. Similarly, regardless of being below or exceeding ST limits, children who were in moderate or high active clusters were more likely to play games with lots of running, play games with some activity, ride a bike/tricycle or scooter, engage in dancing and movement at age 7- 8, compared to the low active group. These results add to the body of literature which report that children can be physically active alongside a combination of other movement behaviours [30, 54]. However, it is important to note, that children in the mixed ST and ST below clusters were most strongly associated with the PA behaviours at age 5 and at age 7- 8. Additionally, children in the ST below cluster were less likely to play on computers or tablet devices at age 5 and at age 7-8. These findings highlight the need to consider the influence of ST on children’s PA behaviours. In their study exploring the simultaneous development of PA, sleep and ST in Canadian children and adolescents, Gallant et al. [55] report that these behaviours are interrelated and that while the majority did not comply with movement guidelines, compliance with PA and/or ST guidelines varied across different sub-groups. Recent research by Dumuid et al. [56] and Fairclough et al. [26] use compositional data analysis to evaluate health predictions of reallocating time between children’s 24 h movement behaviours such as sedentary behaviour (including ST), light PA, MVPA and sleep [26, 56]. These studies analyse behaviours relative to one another to illustrate the relationships between time use and health. Fairclough et al. [26] report that replacing MVPA with any other movement behaviour predicted the most detrimental effects on children’s health, particularly for overweight and obese children. They highlight the need to advocate for the reallocation of time from light PA and sedentary behaviours such as ST to MVPA for health benefits. The findings in the current study illustrate that ST may not influence future PA behaviours directly, but possibly indirectly by reducing the amount of time spent on computers or tablet devices. Further research is necessary to explore the relationship between ST and children’s future PA behaviours. More specifically, investigations to establish thresholds in which one influences the other would help to inform future policy on ST and PA.

The current study highlights the importance of establishing good PA habits in children as young as 3 years of age. All clusters where children engaged in moderate or high levels of PA were more likely to engage in the physical activities reported at age 7/ 8. Since longitudinal studies are difficult to facilitate, there is limited data on the tracking of PA levels in early childhood (age 0-5.9 years) and from early childhood to middle childhood (age 6-11 years). However, two reviews [57, 58] include a total of 12 papers which investigate evidence of PA tracking during these life stages. Jones et al. [57] include 7 papers in their systematic review, all of which measured PA using devices. Of the papers included in the review by Telama [58] five are relevant to this age group. Overall, from these studies, there is evidence of moderate tracking of PA in both early childhood and from early childhood to middle childhood. The evidence of PA tracking from age 3 to age 5 and age 7- 8 in the current study, coupled with that reported by previous literature [57, 58] emphasises the need to establish positive PA behaviours in children as early as possible. This has implications for policy and public health strategies which should target early childhood as a key stage for PA interventions.

In addition, our findings highlight the important role that co-participation in PA - i.e. parents and their children being physically active together - from an early age plays in fostering positive PA behaviours later in childhood. Children who engaged in moderate or high levels of co-participation in PA at age 3 were more likely to attend a sports club/group, climb on tree/frames/wall bars, play with a ball, play chase, ride a bike/tricycle/scooter and skate at age 5 years. A similar impact was seen in engagement in physical activities at age 7 or 8 (play games with a lot of running, play games with some activity, ride a bicycle/tricycle/scooter, and enjoy dancing, music and movement). Based on social-cognitive theory [59] co-participation in PA may be beneficial for PA levels of both parents and children through role modelling of the behaviour (predominantly from parents to children) and/or employing reciprocal reinforcement from both parties to foster active pursuits (parents facilitating children’s PA and vice versa) [51]. Reciprocal reinforcement, where parents are encouraged to role model positive behaviours and become PA advocates for the benefit of their children, and vice versa, has proven to be a successful strategy in parent-child interventions [60–62]. Our findings demonstrate that the benefits of PA co-participation can persist for up to 5 years during childhood.

Gender related differences in daily PA have been noted in children as young as 3 years [63]. There were more boys in the cluster categorised as “High active & Mixed ST” (63% of children in the cluster) and more girls in the cluster categorised as “Low active & ST Exceed” (60%). Our findings demonstrate that the commonly observed gender related differences in PA are seen even when participants are classified into homogenous groups based on categories of PA and ST engagement. Furthermore, there were different patterns in the type of PA engagement for girls and boys. For example, at age 3 boys were more likely to attend a sports club, engage in climbing activities, play with a ball, but less likely than girls to play chase, ride a bike, tricycle or scooter, and engage in skating activities. While at age 7- 8 years, girls were more likely to engage in games with some activity, ride a bicycle, tricycle or scooter and enjoy dance, music and movement, while boys were more likely to play games with a lot of running and play on a computer or tablet device. Gallant et al. [55] reported similar gender-differences in particular behaviours with boys more likely to meet MVPA recommendations while also participating in more ST than girls. Gender differences in PA have been found to persist into adolescence with girls internationally reported to be less active than boys [64]. Additionally, Parker et al. [30] report that mostly adolescent girls composed the ‘non sporty active commuters’ cluster and a significant decline in MVPA was identified for this group in comparison to other groups over time. Given the different patterns of PA engagement between boys and girls and the significant decline in PA for girls over time, the need to prioritise girls for PA promoting interventions, particularly sport engagement is highlighted. It is likely that parents have a great influence on their children’s pattern of engagement, for example through encouragement, social support, involvement, restriction of PA, facilitation such as provision of transportation and sports club enrolment, and role modelling [65].

This study highlights the need to implement PA interventions in early childhood. Children should be provided with opportunities to engage in PA at home, in (pre)school and in community settings. Parents and PCGs should be educated on the importance of promoting and engaging in PA with their child(ren). Parents, PCGs and teachers should be educated on the reallocation of time for health benefits, for example replacing ST behaviours with PA. In (pre)school settings children should be provided with indoor and outdoor play opportunities that incorporate appropriate intensity PA.

The use of clustering analysis in the current study is a novel approach to examine PA and ST behaviours in young children. Since PA and ST have distinct impacts on long term health [66] it is essential to evaluate factors which may influence children’s participation in such behaviours over time. Results of the current study can be used to inform the identification of groups of children most at risk and in need of early PA intervention, since engagement in PA at age 3 predicted future PA engagement. This study includes data from an exceptionally large, nationally representative sample of children which enhances its strength. Moreover, the children are followed through from age 3 to age 7- 8 years and, since longitudinal studies are difficult to facilitate, the use of longitudinal data is an additional strength of this study. However, we acknowledge several limitations. Firstly, the PCG completed the questionnaire on behalf of the child at all three time points. We appreciate that by age 5 and 7- 8, the child may have answered questions about their own behaviour differently. There was also variation in the questions asked at different time points and not all questions have been validated or tested for reliability. Due to unavailable data at Time 3, we were unable to control for the PCG’s age at this timepoint. Furthermore, as is common in longitudinal studies there is some missing data at each time point. We are unable to determine if those who dropped out had the most detrimental changes in PA or other behaviours. Additionally, the duration of follow-up time between measurements was not standardised and this was not accounted for in the analyses. Finally, since there was no validated question used about the child’s PA level (e.g. child equivalent of the International Physical Activity Questionnaire), we could only look at PA-related behaviours in our analysis.

## Conclusions

This longitudinal analysis of over 5,000 children revealed how PA and ST behaviours cluster and the resulting impact on future behaviours. Regardless of ST level, being part of a cluster with moderate or high levels of PA positively influences a child’s future PA at age 5 and again at age 7 or 8 years. Children who engage in moderate or high levels of PA with their parents, are more likely to have active past-times at age 5 and age 7- 8. This paper highlights the vital importance of establishing positive health-related behaviours during early childhood, as this predicts future engagement in health-promoting and health-protective physical activities. The multiple layers of influence on a child’s development, e.g. individual, family, schooling, should be further explored to identify their rolls in supporting the adoption of health-enhancing behaviours.

## Supplementary Information

**Additional file 1.**

**Additional file 2.**

**Additional file 3.**

**Additional file 4.**

## Data Availability

The datasets generated and/or analysed during the current study are available in the Irish Social Science Data Archive (ISSDA), www.ucd.ie/issda.

## Abbreviations

FT: free time
GUI: Growing Up in Ireland
HSE: Health Service Executive
MVPA: moderate-to-vigorous physical activity
OR: odds ratio
PA: physical activity
PCG: primary care giver
ST: recreational screen time
WHO: World Health Organization
