# Supplementary material for: The clustering of physical activity and screen time behaviours in early childhood and impact on future health-related behaviours: a longitudinal analysis of children aged 3 to 8 years
Source: BMC Public Health. 2022 Mar 21;22:558. doi: 10.1186/s12889-022-12944-0 (PMC8939161; doi:10.1186/s12889-022-12944-0)
Supplement: Supplementary file 1 — Additional file 1. [file 12889_2022_12944_MOESM1_ESM.docx]

**Supplementary File 1: GUI Questions with Response Options**

This document outlines the Growing Up in Ireland questionnaire (ESRI, DCU) items relevant to the current study. The response options are also outlined.

**GUI Infant Cohort Wave 2 Questionnaire (Time 1, Age 3)**

Q. On how many days in an average week does anyone at home play active games with <child> (e.g. football)?

0 days 1 day 2 days 3 days 4 days 5 days 6 days 7 days

Q. Typically, how many hours a day does <child> sit and watch television or videos/dvds?

______hours ______minutes [If none, enter 0 for hours and minutes]

Q. What does <child> prefer to do when he/she has a choice about how to spend free time?

Usually chooses inactive pastimes like TV, drawing or playing with toys in one place..

Usually chooses active pastimes like running around, riding push-cars, kicking balls..

Just as likely to choose active as inactive ......................................................................

Q. Can your child ride a tricycle or other similar toy vehicle with pedals?

Yes, can use pedals to cycle ...............................................................................................

Can sit on tricycle and push it along with his/her feet but does not pedal properly yet…. No..................................................................................................................................……

Not sure/doesn’t have tricycle............................................................................................

**GUI Infant Cohort Wave 3 Questionnaire (Time 2, Age 5)**

Q. Does <child> attend a sports club or sports group?

Never…………………………………………………………

Regularly, more than two hours per week…

Regularly, two hours per week…………………..

Regularly, one hour per week…………………….

Twice a month…………………………………………..

Don’t know……………………………………………….

Q. Looking at Card E6, can you tell me how often

|  | Never | Less than once per week | 1-2 times per week | 3-6 times per week | Every day | Don’t know |
| --- | --- | --- | --- | --- | --- | --- |
| a) Climbs on trees, climbing frame, wall bars etc |  |  |  |  |  |  |
| b) Plays with a ball |  |  |  |  |  |  |
| c) Plays chasing |  |  |  |  |  |  |
| d) Rides a bike, tricycle or scooter |  |  |  |  |  |  |
| e) Skates |  |  |  |  |  |  |

|  | Never | Less than once per week | 1-2 times per week | 3-6 times per week | Every day |
| --- | --- | --- | --- | --- | --- |
| Plays on a device like a computer or iPad by themselves |  |  |  |  |  |

**GUI Infant Cohort Wave 4 Questionnaire (Time 3, Age 7/8)**

Q. Can you tell me how often the Study Child takes part in the following activities outside school?

|  | Never | Less than once per week | 1-2 times per week | 3-6 times per week | Every day |
| --- | --- | --- | --- | --- | --- |
| Plays games that involve a lot of running around, like football |  |  |  |  |  |
| Plays games that involve some activity like trampolining |  |  |  |  |  |
| Rides a bike/ tricycle or scooter |  |  |  |  |  |
| Plays on a device like a computer or iPad by themselves |  |  |  |  |  |
| Enjoys dance, music, movement |  |  |  |  |  |
