# Supplementary material for: The clustering of physical activity and screen time behaviours in early childhood and impact on future health-related behaviours: a longitudinal analysis of children aged 3 to 8 years
Source: BMC Public Health. 2022 Mar 21;22:558. doi: 10.1186/s12889-022-12944-0 (PMC8939161; doi:10.1186/s12889-022-12944-0)
Supplement: Supplementary file 2 — Additional file 2. [file 12889_2022_12944_MOESM2_ESM.docx]

Supplementary File 2. Breakdown of participant characteristics within each cluster

|  | Low Active & ST Exceed | High Active & Mixed ST | Mixed activity, No bike & ST Exceed | Mod Active, Active FT & ST Exceed | Mod Active & ST Below | Mod Active & ST Exceed |
| --- | --- | --- | --- | --- | --- | --- |
| Total (%) | 1951 (20) | 2263 (23.2) | 892 (9.1) | 1558 (15.9) | 1139 (11.7) | 1968 (20.1) |
| **Child Gender (%)** |  |  |  |  |  |  |
| Male | 780 (40.0) | 1418 (62.7) | 450 (50.4) | 834 (53.5) | 505 (44.3) | 969 (49.2) |
| Female | 1171 (60.0) | 845 (37.3) | 442 (49.6) | 724 (46.5) | 634 (55.7) | 999 (50.8) |
| **PCG Gender (%)** |  |  |  |  |  |  |
| Male | 28 (1.4) | 40 (1.8) | 11 (1.2) | 25 (1.6) | 15 (1.3) | 40 (2.0) |
| Female | 1923 (98.6) | 2223 (98.2) | 881 (98.8) | 1533 (98.4) | 1124 (98.7) | 1928 (98.0) |
| **PCG Age (%)** |  |  |  |  |  |  |
| 18-29 years | 367 (18.8) | 483 (21.3) | 153 (17.2) | 313 (20.1) | 136 (11.9) | 387 (19.7) |
| 30-39 years | 1279 (65.6) | 1417 (62.6) | 552 (61.9) | 1015 (65.1) | 778 (68.3) | 1258 (63.9) |
| 40+ years | 305 (15.6) | 363 (16.0) | 187 (21.0) | 230 (14.8) | 225 (19.8) | 323 (16.4) |
| **PCG Employment (%)** |  |  |  |  |  |  |
| At Education | 37 (1.9) | 40 (1.8) | 17 (1.9) | 27 (1.7) | 22 (1.9) | 37 (1.9) |
| At work/in training | 1074 (55.0) | 1218 (53.8) | 467 (52.4) | 923 (59.2) | 738 (64.8) | 1037 (52.7) |
| Unemployed | 119 (6.1) | 131 (5.8) | 40 (4.5) | 94 (6.0) | 37 (3.2) | 105 (5.3) |
| Home duties | 685 (35.1) | 827 (36.%) | 343 (38.5) | 484 (31.1) | 322 (28.3) | 736 (37.4) |
| Other | 36 (1.8) | 47 (2.1) | 25 2.8) | 30 (1.9) | 20 (1.8) | 53 (2.7) |

PCG = Primary Care Giver ; ST = Recreational Screen Time; FT = Free Time
