# Supplementary material for: The clustering of physical activity and screen time behaviours in early childhood and impact on future health-related behaviours: a longitudinal analysis of children aged 3 to 8 years
Source: BMC Public Health. 2022 Mar 21;22:558. doi: 10.1186/s12889-022-12944-0 (PMC8939161; doi:10.1186/s12889-022-12944-0)
Supplement: Supplementary file 3 — Additional file 3. [file 12889_2022_12944_MOESM3_ESM.docx]

Table 3. Likelihood of engaging in various activities at age 5, based on demographic characteristics and cluster placement at age 3. Reference category is the Low Active & Recreational Screen Time Exceeding.

|  | **Activity at Time 2** | Attends a sports club or sports group | | | | Climbs on trees, frames, wall bars etc. | | | | Plays with a ball | | | |
| --- | --- | --- | --- | --- | --- | --- | --- | --- | --- | --- | --- | --- | --- |
| **Independent Variables** | Grouping vs Low | Moderate | | High | | Moderate | | High | | Moderate | | High | |
|  | No. included in analysis^ | 6918 | | 6116 | | 4828 | | 6467 | | 2692 | | 6809 | |
|  | χ2 (df) | 129.57 (13)** | | 224.92 (13)** | | 61.84 (13)** | | 345.48 (13)** | | 37.38 (13)** | | 417.66 (13)** | |
|  | Nagelkerke R^2^ | 0.025 | | 0.052 | | 0.017 | | 0.070 | | 0.019 | | 0.112 | |
|  |  | OR | 95% CI | OR | 95% CI | OR | 95% CI | OR | 95% CI | OR | 95% CI | OR | 95% CI |
| Gender of Child | Female | - | - | - | - | - | - | - | - | - | - | - | - |
|  | Male | 1.11* | 1.01-1.23 | 1.43** | 1.27-1.60 | 1.22** | 1.09-1.37 | 1.65** | 1.49-1.83 | 1.40** | 1.17-1.67 | 3.03** | 2.59-3.56 |
| Gender of PCG | Female | - | - | - | - | - | - | - | - | - | - | - | - |
|  | Male | 0.79 | 0.54-1.15 | 0.91 | 0.60-1.38 | 0.84 | 0.55-1.29 | 0.87 | 0.60-1.26 | 0.70 | 0.40-1.23 | 0.76 | 0.46-1.24 |
| Age of PCG | 18-29 years | - | - | - | - | - | - | - | - | - | - | - | - |
|  | 30 – 39 years | 1.34** | 1.13-1.58 | 1.80** | 1.45-2.23 | 1.03 | 0.84-1.25 | 1.09 | 0.92-1.29 | 1.13 | 0.85-1.51 | 0.92 | 0.71-1.19 |
|  | 40+ years | 1.48** | 1.24-1.78 | 1.92** | 1.53-2.41 | 1.21 | 0.99-1.49 | 1.14 | 0.95-1.37 | 1.14 | 0.84-1.55 | 0.84 | 0.64-1.10 |
| Employment Of PCG | Unemployed | - | - | - |  | - | - | - | - | - | - | - | - |
|  | At school/education | 1.12 | 0.69-1.82 | 0.94 | 0.50-1.75 | 0.85 | 0.48-1.48 | 0.68 | 0.42-1.10 | 0.77 | 0.35-1.68 | 0.75 | 0.37-1.52 |
|  | At work or in training | 1.67** | 1.29-2.17 | 1.74** | 1.27-2.37 | 1.27 | 0.94-1.73 | 0.89 | 0.69-1.14 | 0.90 | 0.58-1.40 | 0.81 | 0.54-1.22 |
|  | Home Duties | 1.15 | 0.89-1.49 | 1.20 | 0.87-1.64 | 1.20 | 0.88-1.64 | 0.95 | 0.73-1.23 | 0.77 | 0.49-1.20 | 0.79 | 0.53-1.19 |
|  | Other | 1.17 | 0.79-1.73 | 1.59* | 1.03-2.46 | 1.20 | 0.76-1.88 | 1.02 | 0.70-1.50 | 0.67 | 0.36-1.24 | 0.72 | 0.41-1.25 |
| Cluster Placement at 3 years of age | Low Active & ST Exceed | - | - | - | - | - | - | - | - | - | - | - | - |
|  | High Active & Mixed ST | 1.25** | 1.07-1.46 | 1.55** | 1.29-1.85 | 1.51** | 1.26-1.81 | 2.81** | 2.40-3.30 | 1.37* | 1.04-1.82 | 3.70** | 2.88-4.76 |
|  | Mixed activity, No bike & ST Exceed | 1.00 | 0.82-1.22 | 0.99 | 0.78-1.27 | 0.98 | 0.79-1.22 | 1.15 | 0.94-1.40 | 0.88 | 0.67-1.15 | 0.85 | 0.67-1.09 |
|  | Mod Active, Active FT & ST Exceed | 1.25* | 1.05-1.47 | 1.50** | 1.23-1.82 | 1.39** | 1.15-1.67 | 1.70** | 1.44-2.02 | 1.28 | 0.99-1.67 | 1.85** | 1.46-2.35 |
|  | Mod Active & ST Below | 1.56** | 1.30-1.87 | 2.18** | 1.78-2.68 | 1.29* | 1.05-1.59 | 2.26** | 1.88-2.72 | 1.13 | 0.85-1.50 | 1.98** | 1.54-2.56 |
|  | Mod Active & ST Exceed | 1.18* | 1.01-1.38 | 1.25* | 1.04-1.51 | 1.33** | 1.12-1.58 | 1.74** | 1.49-2.04 | 1.35* | 1.06-1.71 | 1.91** | 1.54-2.38 |

Table 3 cont.

|  | **Activity at Time 2** | Plays Chase | | | | Rides a bike, tricycle or scooter | | | | Skates | | | |
| --- | --- | --- | --- | --- | --- | --- | --- | --- | --- | --- | --- | --- | --- |
| **Independent Variables** | Grouping vs Low | Moderate | | High | | Moderate | | High | | Moderate | | High | |
|  | No. included in analysis^ | 1246 | | 7713 | | 2372 | | 7155 | | 8246 | | 8310 | |
|  | χ2 (df) | 26.66 (13)* | | 83.39 (13)** | | 72.44 (13)** | | 246.572 (13)** | | 94.14 (13)** | | 127.14 (13)** | |
|  | Nagelkerke R^2^ | 0.031 | | 0.037 | | 0.041 | | 0.065 | | 0.039 | | 0.047 | |
|  |  | OR | 95% CI | OR | 95% CI | OR | 95% CI | OR | 95% CI | OR | 95% CI | OR | 95% CI |
| Gender of Child | Female | - | - | - | - | - | - | - | - | - | - | - | - |
|  | Male | 0.42** | 0.33-0.53 | 1.15 | 0.91-1.44 | 0.68** | 0.57-0.81 | 0.77** | 0.66-0.89 | 0.42** | 0.33-0.53 | 0.37** | 0.30-0.46 |
| Gender of PCG | Female | - | - | - | - | - | - | - | - | - | - | - | - |
|  | Male | 0.67 | 0.27-1.68 | 0.64 | 0.33-1.24 | 0.68 | 0.40-1.18 | 0.61* | 0.39-0.96 | 0.67 | 0.27-1.68 | 0.91 | 0.42-1.99 |
| Age of PCG | 18-29 years | - | - | - | - | - | - | - | - | - | - | - | - |
|  | 30 – 39 years | 0.60** | 0.44-0.81 | 0.89 | 0.60-1.33 | 0.87 | 0.64-1.17 | 0.90 | 0.70-1.17 | 0.60** | 0.44-0.81 | 0.78 | 0.57-1.06 |
|  | 40+ years | 0.59** | 0.42-0.83 | 0.79 | 0.52-1.20 | 0.87 | 0.63-1.19 | 0.74* | 057-0.97 | 0.59** | 0.42-0.83 | 0.71* | 0.50-0.99 |
| Employment Of PCG | Unemployed | - | - | - | - | - | - | - | - | - | - | - | - |
|  | At school/education | 0.58 | 0.23-1.46 | 0.51 | 0.18-1.46 | 0.90 | 0.41-2.00 | 0.64 | 0.33-1.25 | 0.58 | 0.23-1.46 | 1.04 | 0.45-2.44 |
|  | At work or in training | 0.47** | 0.31-0.73 | 0.72 | 0.37-1.39 | 1.15 | 0.72-1.82 | 0.89 | 0.60-1.30 | 0.47** | 0.31-0.73 | 0.73 | 0.46-1.17 |
|  | Home Duties | 0.58* | 0.37-0.90 | 0.62 | 0.32-1.21 | 0.85 | 0.53-1.36 | 0.74 | 0.50-1.10 | 0.58* | 0.37-0.90 | 0.84 | 0.52-1.35 |
|  | Other | 0.81 | 0.42-1.57 | 0.46 | 0.20-1.07 | 0.77 | 0.40-1.48 | 0.67 | 0.39-1.15 | 0.81 | 0.42-1.57 | 0.80 | 0.39-1.67 |
| Cluster Placement at 3 years of age | Low Active & ST Exceed | - | - | - | - | - | - | - | - | - | - | - | - |
|  | High Active & Mixed ST | 1.49* | 1.07-2.07 | 2.62** | 1.82-3.78 | 1.41* | 1.06-1.87 | 3.09** | 2.43-3.93 | 1.49* | 1.07-2.07 | 1.43* | 1.06-1.91 |
|  | Mixed activity, No bike & ST Exceed | 0.63 | 0.37-1.07 | 0.69* | 0.49-0.97 | 0.63** | 0.48-0.83 | 0.53** | 0.42-0.67 | 0.63 | 0.37-1.07 | 0.29** | 0.15-0.55 |
|  | Mod Active, Active FT & ST Exceed | 1.27 | 0.88-1.83 | 1.69** | 1.18-2.43 | 1.24 | 0.93-1.64 | 1.86** | 1.46-2.37 | 1.27 | 0.88-1.83 | 1.09 | 0.78-1.53 |
|  | Mod Active & ST Below | 1.06 | 0.70-1.60 | 2.83** | 1.77-4.55 | 1.35 | 0.99-1.83 | 1.79** | 1.37-2.33 | 1.06 | 0.70-1.60 | 0.84 | 0.57-1.23 |
|  | Mod Active & ST Exceed | 1.17 | 0.83-1.65 | 1.89** | 1.34-2.66 | 1.34* | 1.04-1.73 | 1.71** | 1.37-2.13 | 1.17 | 0.83-1.65 | 1.13 | 0.83-1.53 |

|  | **Activity at Time 2** | Plays on computer or tablet | | | |
| --- | --- | --- | --- | --- | --- |
| **Independent Variables** | Grouping vs Low | Moderate | | High | |
|  | No. included in analysis^ | 6208 | | 6184 | |
|  | χ2 (df) | 36.71(13)** | | 134.00(13)** | |
|  | Nagelkerke R^2^ | 0.008 | | 0.029 | |
|  |  | OR | 95% CI | OR | 95% CI |
| Gender of Child | Female | - | - | - | - |
|  | Male | 1.19** | 1.07-1.32 | 1.45** | 1.30-1.60 |
| Gender of PCG | Female | - | - | - | - |
|  | Male | 0.99 | 0.67-1.48 | 1.31 | 0.91-1.89 |
| Age of PCG | 18-29 years | - | - | - | - |
|  | 30 – 39 years | 0.87 | 0.73-1.04 | 0.72** | 0.61-0.86 |
|  | 40+ years | 0.90 | 0.74-1.08 | 0.75** | 0.59-0.85 |
| Employment Of PCG | Unemployed | - | - | - | - |
|  | At school/education | 1.09 | 0.65-1.86 | 1.30 | 0.81-2.09 |
|  | At work or in training | 1.29 | 0.98-1.68 | 0.98 | 0.76-1.26 |
|  | Home Duties | 1.04 | 0.79-1.37 | 0.86 | 0.66-1.11 |
|  | Other | 1.31 | 0.88-1.96 | 1.36 | 0.93-1.98 |
| Cluster Placement at 3 years of age | Low Active & ST Exceed | - | - | - | - |
|  | High Active & Mixed ST | 0.88 | 0.75-1.03 | 1.02 | 0.87-1.19 |
|  | Mixed activity, No bike & ST Exceed | 0.81* | 0.65-0.99 | 1.07 | 0.87-1.30 |
|  | Mod Active, Active FT & ST Exceed | 0.77** | 0.65-0.92 | 0.82* | 0.69-0.98 |
|  | Mod Active & ST Below | 0.81** | 0.68-0.97 | 0.61** | 0.50-0.75 |
|  | Mod Active & ST Exceed | 0.89 | 0.76-1.05 | 1.10 | 0.93-1.29 |

^ = Low category is always included in the analysis; * = <0.05; ** = <0.01; PCG =Primary Care Giver; ST = Recreational Screen Time; FT =Free Time
