# Supplementary material for: The clustering of physical activity and screen time behaviours in early childhood and impact on future health-related behaviours: a longitudinal analysis of children aged 3 to 8 years
Source: BMC Public Health. 2022 Mar 21;22:558. doi: 10.1186/s12889-022-12944-0 (PMC8939161; doi:10.1186/s12889-022-12944-0)
Supplement: Supplementary file 4 — Additional file 4. [file 12889_2022_12944_MOESM4_ESM.docx]

Table 4. Likelihood of engaging in various activities at age 7/8, based on demographic characteristics and cluster placement at age 3. Reference category is the Low Active & Recreational Screen Time Exceeding.

|  | **Activity at Time 3** | Plays games with a lot of running | | | | Plays games with some activity | | | | Rides a bike, tricycle or scooter | | | |
| --- | --- | --- | --- | --- | --- | --- | --- | --- | --- | --- | --- | --- | --- |
| **Independent Variables** | Grouping vs Low | Moderate | | High | | Moderate | | High | | Moderate | | High | |
|  | No. included in analysis^ | 2494 | | 3063 | | 3055 | | 3302 | | 2864 | | 3291 | |
|  | χ2 (df) | 49.74 (10)** | | 194.29 (10)** | | 59.94 (10)** | | 99.02 (10)** | | 42.59 (10)** | | 115.71 (10)** | |
|  | Nagelkerke R^2^ | 0.029 | | 0.096 | | 0.026 | | 0.040 | | 0.020 | | 0.047 | |
|  |  | OR | 95% CI | OR | 95% CI | OR | 95% CI | OR | 95% CI | OR | 95% CI | OR | 95% CI |
| Gender of Child | Female | - | - | - | - | - | - | - | - | - | - | - | - |
|  | Male | 0.98 | 0.81-1.18 | 2.02** | 1.68-2.42 | 0.64** | 0.55-0.74 | 0.69** | 0.60-0.80 | 0.70** | 0.60-0.82 | 0.73** | 0.63-0.84 |
| Gender of PCG | Female | - | - | - | - | - | - | - | - | - | - | - | - |
|  | Male | 1.13 | 0.61-2.10 | 0.92 | 0.50-1.71 | 0.88 | 0.56-1.38 | 0.56* | 0.35-0.90 | 0.76 | 0.47-1.24 | 0.71 | 0.44-1.14 |
| Employment Of PCG | Working | - | - | - | - | - | - | - | - | - | - | - | - |
|  | Training or education | 0.79 | 0.40-1.54 | 0.72 | 0.38-1.39 | 0.78 | 0.46-1.33 | 0.75 | 0.44-1.28 | 1.93* | 1.01-3.69 | 1.99* | 1.05-3.78 |
|  | Home Duties | 0.76** | 0.62-0.93 | 0.74** | 0.60-0.90 | 0.84* | 0.71-0.99 | 1.00 | 0.85-1.17 | 0.94 | 0.76-1.12 | 1.18* | 1.00-1.40 |
|  | Other | 0.76 | 0.55-1.06 | 0.73 | 0.53-1.02 | 0.78 | 0.59-1.04 | 1.16 | 0.89-1.51 | 1.02 | 0.76-1.38 | 1.36* | 1.03-1.80 |
| Cluster Placement at 3 years of age | Low Active & ST Exceed | - | - | - | - | - | - | - | - | - | - | - | - |
|  | High Active & Mixed ST | 1.31 | 0.96-1.77 | 2.91** | 2.16-3.92 | 1.22 | 0.97-1.53 | 2.23** | 1.79-2.78 | 1.49** | 1.17-1.90 | 2.53** | 2.01-3.19 |
|  | Mixed activity, No bike & ST Exceed | 0.55** | 0.40-0.74 | 0.70** | 0.51-0.95 | 0.88 | 0.67-1.15 | 0.95 | 0.72-1.26 | 0.84 | 0.64-1.10 | 0.83 | 0.63-1.10 |
|  | Mod Active, Active FT & ST Exceed | 1.19 | 0.88-1.61 | 1.73** | 1.28-2.34 | 1.07 | 0.84-1.36 | 1.48** | 1.17-1.87 | 1.10 | 0.85-1.41 | 1.74** | 1.36-2.21 |
|  | Mod Active & ST Below | 1.64** | 1.17-2.30 | 2.53** | 1.80-3.55 | 1.42** | 1.10-1.83 | 1.87** | 1.46-2.41 | 1.16 | 0.90-1.50 | 1.40** | 1.09-1.80 |
|  | Mod Active & ST Exceed | 1.02 | 0.79-1.33 | 1.34* | 1.03-1.75 | 1.30* | 1.05-1.62 | 1.47** | 1.18-1.83 | 1.19 | 0.95-1.49 | 1.38** | 1.11-1.73 |

Table 4 cont.

|  | **Activity at Time 3** | Plays on computer or tablet | | | | Dancing or Movement | | | |
| --- | --- | --- | --- | --- | --- | --- | --- | --- | --- |
| **Independent Variables** | Grouping vs Low | Moderate | | High | | Moderate | | High | |
|  | No. included in analysis^ | 2161 | | 3607 | | 2497 | | 3494 | |
|  | χ2 (df) | 56.31 (10)** | | 168.25 (10)** | | 322.04 (10)** | | 928.24(10)** | |
|  | Nagelkerke R^2^ | 0.035 | | 0.069 | | 0.163 | | 0.331 | |
|  |  | OR | 95% CI | OR | 95% CI | OR | 95% CI | OR | 95% CI |
| Gender of Child | Female | - | - | - | - | - | - | - | - |
|  | Male | 1.85** | 1.55-2.22 | 2.51** | 2.13-2.96 | 0.18** | 0.15-0.22 | 0.07** | 0.06-0.09 |
| Gender of PCG | Female | - | - | - | - | - | - | - | - |
|  | Male | 1.20 | 0.64-2.24 | 1.12 | 0.63-1.97 | 1.00 | 0.56-1.77 | 1.22 | 0.71-2.10 |
| Employment Of PCG | Working | - | - | - | - | - | - | - | - |
|  | Training or education | 0.96 | 0.50-1.86 | 0.88 | 0.48-1.58 | 1.74 | 0.85-3.55 | 1.93 | 0.90-3.69 |
|  | Home Duties | 0.95 | 0.78-1.16 | 0.94 | 0.79-1.13 | 0.89 | 0.73-1.09 | 1.09 | 0.90-1.32 |
|  | Other | 0.91 | 0.70-1.38 | 1.05 | 0.78-1.43 | 1.01 | 0.72-1.43 | 1.37 | 0.98-1.90 |
| Cluster Placement at 3 years of age | Low Active & ST Exceed | - | - | - | - | - | - | - | - |
|  | High Active & Mixed ST | 0.97 | 0.74-1.27 | 0.96 | 0.75-1.23 | 1.42** | 1.09-1.86 | 1.73** | 1.33-2.25 |
|  | Mixed activity, No bike & ST Exceed | 1.04 | 0.72-1.49 | 1.22 | 0.88-1.68 | 0.86 | 0.61-1.22 | 1.34 | 0.96-1.86 |
|  | Mod Active, Active FT & ST Exceed | 0.92 | 0.68-1.23 | 0.95 | 0.73-1.24 | 1.27 | 0.96-1.69 | 1.22 | 0.91-1.63 |
|  | Mod Active & ST Below | 0.78 | 0.59-1.04 | 0.64** | 0.49-0.83 | 1.13 | 0.83-1.54 | 1.58** | 1.16-2.15 |
|  | Mod Active & ST Exceed | 1.25 | 0.94-1.66 | 1.37* | 1.06-1.76 | 1.33* | 1.01-1.74 | 1.54** | 1.17-2.01 |

^ = Low category is always included in the analysis; * = <0.05; ** = <0.01; ^ = Moderate/ High + Low category since low category is always included in the analysis; PCG =Primary Care Giver; ST = Recreational Screen Time; FT = Free Time
